# Supplementary material for: Comparative sequence analysis elucidates the evolutionary patterns of Yersinia pestis in New Mexico over thirty-two years
Source: PeerJ. 2023 Sep 26;11:e16007. doi: 10.7717/peerj.16007 (PMC10541020; doi:10.7717/peerj.16007)
Supplement: Supplemental Information 1 — Novel New Mexico Genome Sequence Identifier, SSR number, genome name, year of Isolation, known host, and regional location. [file peerj-11-16007-s001.docx]

| **Sequence ID** | **Sequence Read Archive Identifier** | **Genome Name** | **Year of Isolation** | **Isolation Host** | **Region** |
| --- | --- | --- | --- | --- | --- |
| 2013030697 | SRR18962121 | 0697 | 2013 | Human | Torrance |
| AS20090813 | SRR18962104 | 0813 | 2009 | Cat | Espanola |
| 2015021120-b | SRR18962112 | 1120 | 2015 | Human | Unknown |
| AS200801250 | SRR18962105 | 1250 | 2008 | Cat | Unknown |
| 83-1302a | SRR18962125 | 1302 | 1983 | Human | Unknown |
| AS1546 | SRR18962107 | 1546 | 2003 | Cat | Unknown |
| 1591 | SRR18962106 | 1591 | 2003 | Cat | Unknown |
| 83-1880a | SRR18962124 | 1880 | 1983 | Human | Unknown |
| 88-2060 | SRR18962110 | 2060 | 1988 | Human | Unknown |
| AS200902149 | SRR18962123 | 2149 | 2009 | Unknown | Santa Fe |
| 98-2456 | SRR18962108 | 2456 | 1998 | Human | Unknown |
| 91-3365 | SRR18962109 | 3365 | 1992 | Human | Unknown |
| 88-3385 | SRR18962113 | 3385 | 1988 | Cat | Unknown |
| 2015023558-B | SRR18962114 | 3558 | 2015 | Unknown | Unknown |
| 2014013957 | SRR18962116 | 3957 | 2014 | Human | Unknown |
| 201414290 | SRR18962115 | 4290 | 2014 | Cat | Unknown |
| 2015026020-b | SRR18962111 | 6020 | 2015 | Unknown | Unknown |
| 2013027498 | SRR18962120 | 7498 | 2013 | Cat | Santa Fe |
| 2013027658 | SRR18962119 | 7658 | 2013 | Unknown | Unknown |
| 2013027979 | SRR18962118 | 7979 | 2013 | Cat | Santa Fe |
| 2014028180-b | SRR18962117 | 8180 | 2014 | Unknown | Unknown |
| 2011019706 | SRR18962122 | 9706 | 2011 | Human | Santa Fe |
