# Supplementary material for: Comparative sequence analysis elucidates the evolutionary patterns of Yersinia pestis in New Mexico over thirty-two years
Source: PeerJ. 2023 Sep 26;11:e16007. doi: 10.7717/peerj.16007 (PMC10541020; doi:10.7717/peerj.16007)
Supplement: Supplemental Information 2 — Read coverage depth and breadth of each isolates chromosome. [file peerj-11-16007-s002.docx]

| **Sequence ID** | **Depth of Coverage** | **Breadth of Coverage** |
| --- | --- | --- |
| 2013030697 | 37.7 | 0.999985818 |
| AS20090813 | 49.8 | 0.999964545 |
| AS200801205 | 61.33 | 0.999961751 |
| AS1546 | 57.6 | 0.999995917 |
| 1591 | 69.28 | 0.999990545 |
| 83-1880a | 9.5 | 0.997247583 |
| 88-2060 | 69.56 | 0.999438515 |
| AS200902149 | 49 | 0.99995552 |
| 98-2456 | 20.5 | 0.999982595 |
| 91-3365 | 38.2 | 0.999989256 |
| 88-3385 | 71.4 | 0.999993554 |
| 2014013957 | 54 | 0.999931023 |
| 201414290 | 19.9 | 0.999868278 |
| 2015026020-b | 11.3 | 0.99994499 |
| 2013027498 | 55.38 | 0.999975289 |
| 2013027658 | 33.9 | 0.999986033 |
| 2013027979 | 49.2 | 0.999990975 |
| 2011019706 | 27.3 | 0.99982788 |
| 2015021120-b | 36.7 | 0.999911899 |
| 83-1302a | 38.57 | 0.999976793 |
| 2015023558-B | 26.11 | 0.999980661 |
| 2014028180-b | 28.29 | 0.999976363 |
