# Supplementary material for: Comparative sequence analysis elucidates the evolutionary patterns of Yersinia pestis in New Mexico over thirty-two years
Source: PeerJ. 2023 Sep 26;11:e16007. doi: 10.7717/peerj.16007 (PMC10541020; doi:10.7717/peerj.16007)
Supplement: Supplemental Information 3 — Depth of read coverage for the pPCP, pCD, and pMT plasmids. [file peerj-11-16007-s003.docx]

| **Sequence ID** | **pPCP** | **pCD** | **pMT** |
| --- | --- | --- | --- |
| 2013030697 | 952 | 213 | 488 |
| AS20090813 | 1545 | 238 | 174 |
| 2015021120-b | 420 | 166 | 109 |
| AS200801205 | 4178 | 325 | 228 |
| 83-1302a | 1861 | 260 | 139 |
| AS1546 | 1443 | 246 | 204 |
| 1591 | 1709 | 274 | 251 |
| 83-1880a | 192 | 39 | 28 |
| 88-2060 | 1528 | 260 | 227 |
| AS200902149 | 1719 | 319 | 174 |
| 98-2456 | 434 | 84 | 64 |
| 91-3365 | 785 | 146 | 132 |
| 88-3385 | 2165 | 302 | 226 |
| 2015023558-B | 973 | 160 | 97 |
| 2014013957 | 1720 | 339 | 179 |
| 201414290 | 539 | 106 | 69 |
| 2015026020-b | 169 | 59 | 28 |
| 2013027498 | 1680 | 309 | 187 |
| 2013027658 | 677 | 219 | 302 |
| 2013027979 | 1662 | 302 | 174 |
| 2014028180-b | 701 | 125 | 90 |
| 2011019706 | 506 | 84 | 70 |
