# Supplementary material for: Comparative sequence analysis elucidates the evolutionary patterns of Yersinia pestis in New Mexico over thirty-two years
Source: PeerJ. 2023 Sep 26;11:e16007. doi: 10.7717/peerj.16007 (PMC10541020; doi:10.7717/peerj.16007)
Supplement: Supplemental Information 4 — Percent breadth of read coverage for the pPCP, pCD, and pMT plasmids. [file peerj-11-16007-s004.docx]

| **Sequence ID** | **pPCP** | **pCD** | **pMT** |
| --- | --- | --- | --- |
| 2013030697 | 100 | 100 | 100 |
| AS20090813 | 100 | 100 | 100 |
| 2015021120-b | 100 | 100 | 100 |
| AS200801205 | 100 | 100 | 100 |
| 83-1302a | 100 | 100 | 100 |
| AS1546 | 100 | 100 | 100 |
| 1591 | 100 | 100 | 100 |
| 83-1880a | 100 | 99.99004 | 100 |
| 88-2060 | 100 | 100 | 100 |
| AS200902149 | 100 | 100 | 100 |
| 98-2456 | 100 | 99.99858 | 100 |
| 91-3365 | 100 | 100 | 100 |
| 88-3385 | 100 | 100 | 100 |
| 2015023558-B | 100 | 100 | 100 |
| 2014013957 | 100 | 100 | 100 |
| 201414290 | 100 | 100 | 100 |
| 2015026020-b | 100 | 100 | 99.98857 |
| 2013027498 | 100 | 100 | 100 |
| 2013027658 | 100 | 100 | 100 |
| 2013027979 | 100 | 100 | 100 |
| 2014028180-b | 100 | 100 | 100 |
| 2011019706 | 100 | 99.99858 | 100 |
