# Supplementary material for: Comparative sequence analysis elucidates the evolutionary patterns of Yersinia pestis in New Mexico over thirty-two years
Source: PeerJ. 2023 Sep 26;11:e16007. doi: 10.7717/peerj.16007 (PMC10541020; doi:10.7717/peerj.16007)
Supplement: Supplemental Information 5 — Meta-CATS hits with p-values < 0.017 are shown. CO92 was the reference genome for positions in the first column. Numbers in parenthesis indicate the total number of genomes with that specific variation. The known functions for that region are recorded. [file peerj-11-16007-s005.docx]

| Position | P-Value | 1983-1988 | 1993-1998 | 2003-2009 | 2011-2015 | Function |
| --- | --- | --- | --- | --- | --- | --- |
| 1330310 | 0.0016618 | Guanine (4) | Thymine (2) | Guanine(4), Thymine(1) | Guanine (11) | Non-coding region |
| 1410394 | 0.0016618 | Guanine (4) | Adenine (2) | Guanine(4), Adenine(1) | Guanine (11) | Non-coding region |
| 1552580 | 0.0016618 | Thymine (4) | Guanine (2) | Guanine(4), Thymine(1) | Thymine (11) | tRNA |
| 1846771 | 0.0016618 | Guanine (4) | Gap '-' (2) | Guanine(4), Gap '-'(1) | Guanine (11) | Transcription repair coupling |
| 1846772 | 0.0016618 | Cytosine (4) | Gap '-' (2) | Cytosine(4), Gap '-'(1) | Cytosine (11) | Transcription repair coupling |
| 1846773 | 0.0016618 | Adenine (4) | Gap '-' (2) | Adenine(4), Gap '-'(1) | Adenine (11) | Transcription repair coupling |
| 1846774 | 0.0016618 | Thymine (4) | Gap '-' (2) | Thymine(4), Gap '-'(1) | Thymine (11) | Transcription repair coupling |
| 1846775 | 0.0016618 | Thymine (4) | Gap '-' (2) | Thymine(4), Gap '-'(1) | Thymine (11) | Transcription repair coupling |
| 1846776 | 0.0016618 | Adenine (4) | Gap '-' (2) | Adenine(4), Gap '-'(1) | Adenine (11) | Transcription repair coupling |
| 1956259 | 0.0016618 | Cytosine (4) | Thymine (2) | Cytosine(4), Thymine(1) | Cytosine (11) | Oligogalacturonate lyase product |
| 2647532 | 0.0016618 | Adenine (4) | Gap '-' (2) | Adenine(4), Gap '-'(1) | Adenine (11) | Peptide ABC transporter *SapA* |
| 2647533 | 0.0016618 | Cytosine (4) | Gap '-' (2) | Cytosine(4), Gap '-'(1) | Cytosine (11) | Peptide ABC transporter *SapA* |
| 2647534 | 0.0016618 | Adenine (4) | Gap '-' (2) | Adenine(4), Gap '-'(1) | Adenine (11) | Peptide ABC transporter *SapA* |
| 2647535 | 0.0016618 | Thymine (4) | Gap '-' (2) | Thymine(4), Gap '-'(1) | Thymine (11) | Peptide ABC transporter *SapA* |
| 2647536 | 0.0016618 | Cytosine (4) | Gap '-' (2) | Cytosine(4), Gap '-'(1) | Cytosine (11) | Peptide ABC transporter *SapA* |
| 2647537 | 0.0016618 | Adenine (4) | Gap '-' (2) | Adenine(4), Gap '-'(1) | Adenine (11) | Peptide ABC transporter *SapA* |
| 2647538 | 0.0016618 | Cytosine (4) | Gap '-' (2) | Cytosine(4), Gap '-'(1) | Cytosine (11) | Peptide ABC transporter *SapA* |
| 3439436 | 0.0016618 | Gap '-' (4) | Guanine (2) | Guanine(4), Gap '-'(1) | Gap '-' (11) | Ferric iron ABC transporter |
| 3439437 | 0.0016618 | Gap '-' (4) | Guanine (2) | Guanine(4), Gap '-'(1) | Gap '-' (11) | Ferric iron ABC transporter |
| 3439438 | 0.0016618 | Gap '-' (4) | Guanine (2) | Guanine(4), Gap '-'(1) | Gap '-' (11) | Ferric iron ABC transporter |
| 4358821 | 0.0016618 | Guanine (4) | Adenine (2) | Guanine(4), Adenine(1) | Guanine (11) | Non-coding region |
| 4384651 | 0.0016618 | Cytosine (4) | Adenine (2) | Adenine(4), Cytosine(1) | Cytosine (11) | 5S-rRNA |
| 4584335 | 0.0016618 | Guanine (4) | Adenine (2) | Guanine(4), Adenine(1) | Guanine (11) | Putative Protein |
| 796168 | 0.0025688 | Adenine (4) | Gap '-' (2) | Adenine (5) | Adenine(10), Gap '-'(1) | Flagellin *FliC* |
| 796169 | 0.0025688 | Guanine (4) | Gap '-' (2) | Guanine (5) | Guanine(10), Gap '-'(1) | Flagellin *FliC* |
| 796170 | 0.0025688 | Guanine (4) | Gap '-' (2) | Guanine (5) | Guanine(10), Gap '-'(1) | Flagellin *FliC* |
| 796171 | 0.0025688 | Guanine (4) | Gap '-' (2) | Guanine (5) | Guanine(10), Gap '-'(1) | Flagellin *FliC* |
| 796172 | 0.0025688 | Guanine (4) | Gap '-' (2) | Guanine (5) | Guanine(10), Gap '-'(1) | Flagellin *FliC* |
| 796173 | 0.0025688 | Guanine (4) | Gap '-' (2) | Guanine (5) | Guanine(10), Gap '-'(1) | Flagellin *FliC* |
| 796174 | 0.0025688 | Guanine (4) | Gap '-' (2) | Guanine (5) | Guanine(10), Gap '-'(1) | Flagellin *FliC* |
| 796175 | 0.0025688 | Thymine (4) | Gap '-' (2) | Thymine (5) | Thymine(10), Gap '-'(1) | Flagellin *FliC* |
| 796176 | 0.0025688 | Adenine (4) | Gap '-' (2) | Adenine (5) | Adenine(10), Gap '-'(1) | Flagellin *FliC* |
| 190085 | 0.0065459 | Gap '-' (4) | Gap '-' (2) | Guanine(4), Gap '-'(1) | Guanine(10), Gap '-'(1) | *ArgD* |
| 190086 | 0.0065459 | Gap '-' (4) | Gap '-' (2) | Adenine(4), Gap '-'(1) | Adenine(10), Gap '-'(1) | *ArgD* |
| 190087 | 0.0065459 | Gap '-' (4) | Gap '-' (2) | Adenine(4), Gap '-'(1) | Adenine(10), Gap '-'(1) | *ArgD* |
| 190088 | 0.0065459 | Gap '-' (4) | Gap '-' (2) | Adenine(4), Gap '-'(1) | Adenine(10), Gap '-'(1) | *ArgD* |
| 190089 | 0.0065459 | Gap '-' (4) | Gap '-' (2) | Guanine(4), Gap '-'(1) | Thymine(10), Cytosine(1) | *ArgD* |
| 190090 | 0.0065459 | Gap '-' (4) | Gap '-' (2) | Cytosine(4), Gap '-'(1) | Cytosine(1), Gap '-'(10) | *ArgD* |
| 3081850 | 0.0098924 | Adenine(3), Guanine(1) | Guanine (2) | Guanine(4), Adenine(1) | Adenine(10), Guanine(1) | Hypothetical protein |
| 1391256 | 0.0126378 | Cytosine(3), Thymine(1) | Thymine (2) | Thymine (5) | Thymine(10), Cytosine(1) | Hypothetical protein |
| 961371 | 0.0164831 | Guanine (4) | Guanine (2) | Guanine (5) | Guanine(4), Gap '-'(17) | Hypothetical protein |
| 961372 | 0.0164831 | Adenine (4) | Adenine (2) | Adenine (5) | Adenine(4), Gap '-'(7) | Hypothetical protein |
