# Supplementary material for: Comparative sequence analysis elucidates the evolutionary patterns of Yersinia pestis in New Mexico over thirty-two years
Source: PeerJ. 2023 Sep 26;11:e16007. doi: 10.7717/peerj.16007 (PMC10541020; doi:10.7717/peerj.16007)
Supplement: Supplemental Information 6 — GenBank accession number, genome ID, genome names, collection data, and known isolation country. Genome IDs and GenBank Acession numbers are used for Bayesian tree reconstruction. [file peerj-11-16007-s006.docx]

| **GenBank Accession** | **Genome ID** | **Genome Names** | **Collection Date** | **Isolation Country** |
| --- | --- | --- | --- | --- |
| CP009840.1 | 1035377.9 | Yersinia pestis A1122 | 1939 | USA |
| CP009973.1 | 214092.181 | Yersinia pestis CO92 | 1992 | USA |
| CP009704.1 | 632.126 | Yersinia pestis Harbin35 | 1940 | China |
| CP009723.1 | 632.127 | Yersinia pestis Shasta | 1954 | USA |
| CP009785.1 | 632.128 | Yersinia pestis El Dorado | 2002 | USA |
| CP009844.1 | 632.129 | Yersinia pestis Dodson | 1967 | USA |
| CP064125 | 632.152 | Yersinia pestis strain M2085 | 2015 | Russia |
| CP016273.1 | 632.175 | Yersinia pestis strain Cadman | 2016 | USA |
| CP002956.1 | 632.423 | Yersinia pestis strain FDAARGOS_602 | 1984 | USSR |
| CP063303.2 | 632.704 | Yersinia pestis 14D | 1970 | Russia |
| CP045640.1 | 632.705 | Yersinia pestis S19960127 | 1996 | China |
| CP064123.1 | 632.710 | Yersinia pestis M2029 | 2018 | Russia |
| CP009935.1 | 632.711 | Yersinia pestis C-783 | 2001 | Russia |
| CP064117 | 632.712 | Yersinia pestis strain C-781 | 2001 | Russia |
| CP064128 | 632.713 | Yersinia pestis strain M2086 | 2015 | Russia |
| CP064124.1 | 632.714 | Yersinia pestis M-1974 | 2012 | Russia |
| CP064126.1 | 632.715 | Yersinia pestis I-1252 | 1966 | Russia |
| CP064121.1 | 632.717 | Yersinia pestis M-1482 | 1989 | Russia |
| CP064122.2 | 632.718 | Yersinia pestis M-1770 | 2002 | Russia |
| CP064125.2 | 632.719 | Yersinia pestis M2085 | 2015 | Russia |
| CP064127.1 | 632.720 | Yersinia pestis C-830 | 2012 | Russia |

Supplementary Table 6: Sequence Metadata for PATRIC Database Sequences
