# Supplementary material for: Comparative sequence analysis elucidates the evolutionary patterns of Yersinia pestis in New Mexico over thirty-two years
Source: PeerJ. 2023 Sep 26;11:e16007. doi: 10.7717/peerj.16007 (PMC10541020; doi:10.7717/peerj.16007)
Supplement: Supplemental Information 7 — The genomes 0697, 0813, were isolated from Torrance, and Rio Arriba (Espanola). While genomes 2149, 7498, 7979, and 9706, were isolated from Santa Fe county. Image adapted from https://suncatcherstudio.com. [file peerj-11-16007-s007.pdf]

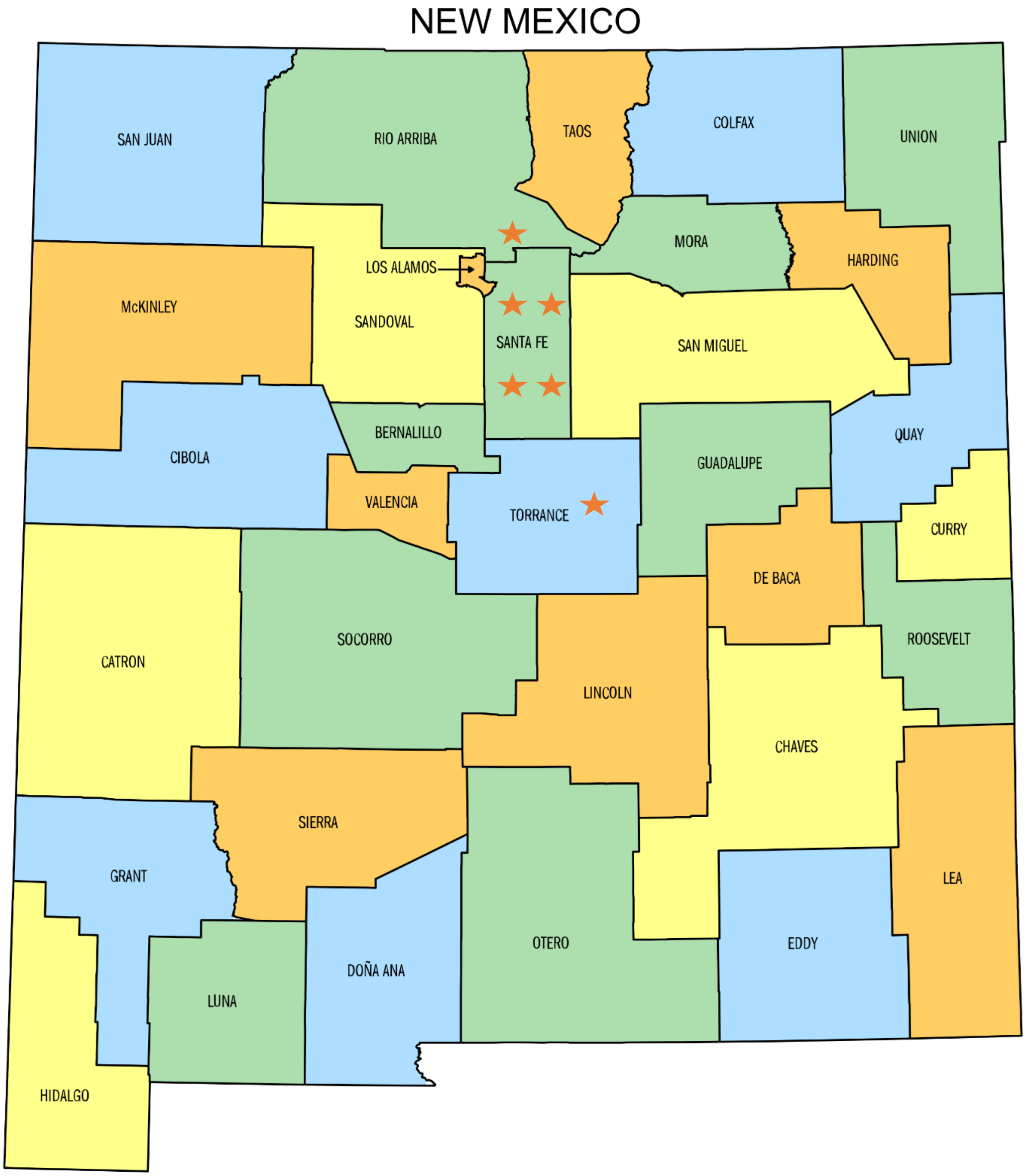

Supplementary Figure 1: **County level data for six novel *Yersinia pestis* isolates.** The genomes 0697, 0813, were isolated from Torrance, and Rio Arriba (Espanola). While genomes 2149, 7498, 7979, and 9706, were isolated from Santa Fe county. Image adapted from https://suncatcherstudio.com.
